# Liquefaction of Cellulose for Production of Advanced Porous Carbon Materials

**DOI:** 10.3390/polym14081621

**Published:** 2022-04-16

**Authors:** Arjeta Kryeziu, Václav Slovák, Alžběta Parchaňská

**Affiliations:** 1 Department of Chemistry, University of Ostrava, 30. Dubna 22, 701 03 Ostrava, Czech Republic; vaclav.slovak@osu.cz (V.S.); alzbeta.parchanska@osu.cz (A.P.); 2 Institut de Science des Matériaux de Mulhouse (IS2M), UMR 7361 CNRS-UHA, Université de Haute-Alsace, 15 Rue Jean Starcky, 68057 Mulhouse, France

**Keywords:** liquefaction, cellulose, dissolution, carbon

## Abstract

Cellulose is a renewable resource for the production of advanced carbonaceous materials for various applications. In addition to direct carbonization, attention has recently been paid to the preparation of porous carbons from liquid cellulose-based precursors. Possible pathways of cellulose conversion to a liquid state suitable for the preparation of porous carbons are summarized in this review. Hydrothermal liquefaction leading to liquid mixtures of low-molecular-weight organics is described in detail together with less common decomposition techniques (microwave or ultrasound assisted liquefaction, decomposition in a strong gravitation field). We also focus on dissolution of cellulose without decomposition, with special attention paid to dissolution of nonderivatized cellulose. For this purpose, cold alkalines, hot acids, ionic liquids, or alcohols are commonly used.

## 1. Introduction

Cellulose, the most abundant natural organic polymer [1], is the main structural component of plant cell walls, as well as many algae and oomycetes. It is the most abundant homopolysaccharide in nature (approximately 1.5 × 10^12^ tons of annual production), a traditional renewable energy source [2,3,4], reserve of carbon and hydrogen, and a raw material for paper making and other industrial processes such as cosmetics, food [5], and medicine production [6,7]. Being a composed of many linear β-(1,4)-linked homopolymer chains of hundreds to thousands of anhydroglucopyranose residues, connected parallel by hydrogen bonds to form tough and inflexible structures, cellulose is a promising source of glucopyranose units and their derivatives as well as mixtures of various low-molecular-weight compounds such as phenolic compounds, furan derivatives, carbonyl compounds or carboxyl compounds, called bio-oil [8]. These small molecules can be further used as precursors for organic syntheses or preparation of various advanced carbonaceous materials such as carbon aerogels with a certain porosity [9]. For effective preparation of the latter materials, it is important to convert the precursors to solution/liquid state since this allows higher versatility in nanoporous carbon preparation. However, the conversion of cellulose macromolecules to this state is complicated since cellulose is insoluble in a majority of common solvents (water, organic solvents) under normal conditions and the β-glycoside bonds that connect the cellulose units can be hydrolyzed by cellulase enzymes [10] or acids [11].

Several procedures for converting solid-state cellulose into a liquid state (i.e., into a solution of cellulose derivatives, a solution of single glucopyranose chains, oligomers or monomers, or bio-oil (a liquid product)) have been developed. The first developed procedures were based on the preparation of soluble cellulose derivatives such as cellulose nitrate, acetate [12], formate [13], carbamate or xanthogenate, the latter being an intermediate in traditional viscose processes [14]. Another traditional treatment of cellulose is its dissolving in cuprammonium hydroxide solution that forms a copper-containing cellulose complex [15], which was later replaced by dissolution in N-methylmorpholine-N-oxide (NMMO) [16,17].

In recent decades, new approaches and procedures for the dissolution of cellulose or conversion of cellulose to a liquid mixture of low-molecular-weight compounds have been invented. Due to the practical insolubility of cellulose under normal conditions [18], procedures usually involve low [19] or high temperatures [20] or high pressure (supercritical conditions) [21], aggressive solvents (concentrated mineral acid) [22], solvent mixtures [23] or other special conditions (microwave heating [24], strong gravitation field, ultrasonic field [25]). All these parameters strongly influence the yield and composition of the obtained liquid mixture.

This review summarizes the procedures for the liquefaction/dissolution of cellulose, resulting in liquid mixtures with a composition appropriate for preparation of nanoporous carbon material with distinct porous properties (specific pore size distribution and specific surface area).

## 2. Processes Based on Cellulose Decomposition

**Figure 1:**
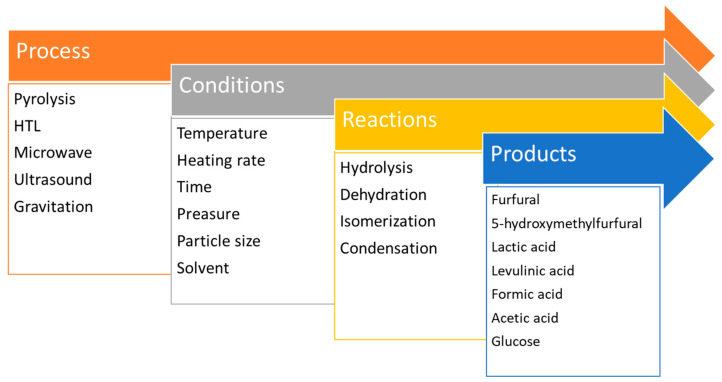
Schematic representation of biomass/cellulose liquefactions.

Biomass or cellulose decomposition to produce a liquid mixture of organic compounds (bio-oil) is in the literature commonly called liquefaction. In general, it is based on the chemical degradation of cellulose chains to low-molecular-weight products. In addition to simple biomass pyrolysis, hydrothermal liquefaction (HTL) and some other techniques enabling the conversion of cellulose to chemicals as carbon precursors are used (Figure 1).

### 2.1. Biomass Pyrolysis

Biomass pyrolysis is a complex process determined by biomass feedstock and reaction parameters. Basic pyrolysis of biomass is carried out at atmospheric pressure. According to the heating rate during pyrolysis, slow, fast and flash pyrolysis are used [26,27]. Three processes are considered during biomass pyrolysis, including char formation, depolymerization, and fragmentation. The major products from biomass pyrolysis are char, bio-oil, and pyrolytic gases.

Char is a solid product with high carbon content; bio-oil is an organic mixture of different compounds such as alcohols, sugars, and other organic compounds containing oxygen; pyrolytic gases contain mostly carbon dioxide, carbon monoxide, hydrogen, and light hydrocarbons [28]. Char, bio-oil, and gases are usually in the range of 13–25, 50–70, and 12–15 wt%. The composition of pyrolysis products and their proportions are influenced by the type of biomass, physical and chemical treatments, pyrolysis temperature, carrier gas, and heating rate. The heating rate is considered as the main factor affecting the yield of bio-oil. During slow pyrolysis (heating rate below 1 K/s) the bio-oil yield is decreased down to about 30 wt%, while increasing the heating rate (fast pyrolysis 10–200 K/s, flash pyrolysis with more than 1000 K/s) enables obtaining more than 70 wt% of bio-oil [27].

**Figure 2:**
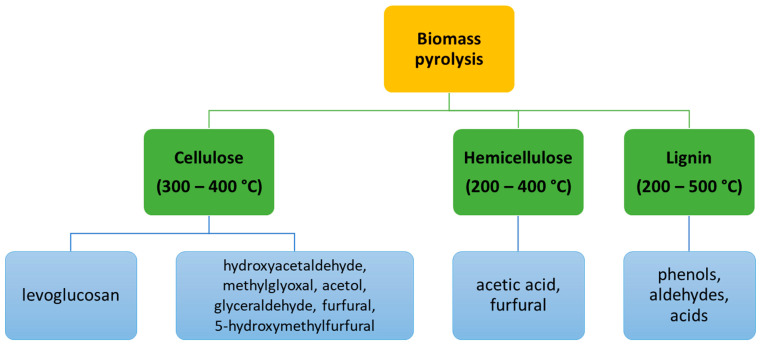
Major components of pyrolytic bio-oil [29].

The composition of pyrolysis bio-oil is related to the decomposition of the main biomass components (Figure 2). In addition to the main constituents, bio-oil contains hundreds (thousands in some of the literature [29]) of compounds (mostly oxygenated) and undergoes significant changes in composition and physical properties during storage (evaporation of volatile components, continuous reactions in the mixture) [27].

### 2.2. Hydrothermal Cellulose Liquefaction (HTL)

Hydrothermal liquefaction (HTL) is a thermochemical processing technology for the production of bio-oil from agricultural and forestry wastes. Bio-oil is a dark, viscous, and energy-dense liquid which has a 70–95% energy content of petroleum fuel oil [30]. The HTL process is based on the decomposition of biomass in hot pressurized water or various organic solvents (alcohols, polyalcohols such as ethyleneglycol [4], or acetone) [31] in the presence of homogeneous catalysts such as sulfuric acid [4], alkali hydroxides, ammonia or inorganic salts such as sodium or potassium carbonate [32], or heterogeneous catalysts such as transition metals or zeolites [33]. Some HTL processes take place under subcritical conditions, while the others are supercritical. As the HTL process does not require prior drying of biomass [34], it can be applied to both dry (lignocellulose) and wet biomass (algae) [35].

**Figure 3:**
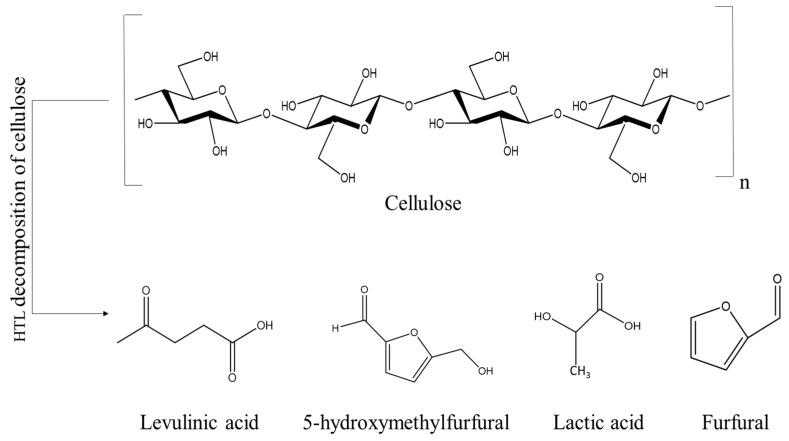
Decomposition products during cellulose liquefaction.

The reactions that occur with cellulose and hemicelluloses in the course of HTL have been thoroughly investigated. It has been found that the key reactions in HTL are hydrolysis, dehydration, isomerization, and retro-aldol condensation. Cellulose and hemicellulose (a mixture of highly branched low-molecular-weight homo and heteropolymers comprised of anhydro-β-(1-4)-D-xylopyranose, glucopyranose, mannopyranose and galactopyranose units [36]) are initially hydrolyzed into oligosaccharides and monosaccharides that are further decomposed into small molecules such as furfural, 5-hydroxymethylfurfural, lactic acid, levulinic acid, formic acid or acetic acid [32,37] (Figure 3). Using ethylene glycol as a solvent for liquefaction, the reaction leads to decomposition of glucosides into glucose [4], organic acids, and polymerized alcohols [38].

The yield and composition of bio-oil depend on the biomass composition: the maximum bio-oil yield is obtained from biomass with a high lignin content [38].

### 2.3. Effect of Processing Parameters on Bio-Oil Yield of HTL

The HTL process is significantly affected by many processing conditions; its appropriate setup is crucial for increasing the yield of bio-oil and its composition.

Temperature is a sensitive operating parameter in the hydrothermal liquefaction process. Cellulose liquefaction occurs through the cleavage of C-C and C-O bonds under the action of solvent radicals produced at high temperature [39]. The temperature usually ranges between 250 and 400 °C and the pressure is set between 5 and 20 MPa. The procedure can take various times, from 10 min up to 24 h [31]. However, after reaching the maximum bio-oil yield at certain temperatures, further increases in temperature lead to decrease of bio-oil yield due to repolymerization of free radicals produced from liquefaction products and due to increased gas production [38]. The biomass type [38] and the solvent type also affect the optimal liquefaction temperature [31]. The increase in cellulose crystallinity results in a change of the optimal liquefaction temperature to approximately 380 °C [40]. Beyond a certain threshold, further increase in temperature or residential time leads to decreased bio-oil yield. This threshold is different for different biomass sources/composition [38].

In general, increasing residence time leads to the increase of bio-oil yields [41]. With the increase in reaction time from the beginning of reaction up to 30 min, the yield of solid residue yield decreased from 86.3% to 60.9%, while the yield of bio-oil increased from 8.7% to 28.5%, due to the deeper liquefaction of cellulose by prolonging the reaction time [42]. At 350 °C, the cellulose liquefaction is carried out with a residence time of 30 min or 1 h. With increasing residence time for more than 1 h, the yields of oil and aqueous phase yields decreased, and only the gas yield increased [43].

Cellulose conversion and its product distribution are significantly affected by the thermal effect [44]. However, the heating rate showed a marginal influence on the bio-oil yield in supercritical EtOH-based liquefaction [45]. The significant effect on biomass conversion is due to the heating rate above 280 °C using ethanol [46,47].

The major difference between liquefaction and pyrolysis experiments lies in the effect of high pressure, which helps to condensate the reaction products and prevents their escape from the reaction vessel. Cellulose is highly oxygenated, and a reducing gas is frequently used to remove oxygen. It is preferable to eliminate oxygen autonomously either as water (which is undesirable since the end product would be carbon) or as carbon dioxide [48]. Higher temperatures will require higher pressure to maintain a liquid water phase [49]. Increasing the reaction pressure is beneficial for the hydrogenation reaction [50] and leads to slowing down of the dissociation reactions, although the effects of solvation may override it. Experiments have shown that when cellulose is heated to 350 °C under pressure, and in the presence of water, phenol and certain catalysts such as oxalic acid or sulfuric acid, almost total liquefaction can occur [51].

Biomass feedstock is one of the important parameters that affect bio-oil yield. The high cellulose content in biomass leads to high bio-oil yield [52].

Optimum size of biomass particles increases the yield of bio-oil at low grinding cost since reduction in particle size improves heat transfer and increases surface area [53]. Usually, particle diameters between 4 and 10 mm are suitable to overcome heat and mass transfer at reasonable grinding cost [52].

### 2.4. Solvents in HTL

A very important and key factor [54] for the efficiency of the liquefaction process and the properties of the final product is the selection of the appropriate solvent [55] (Table 1). The choice of solvent can have a strong impact on the distribution and yield [56]. The switch from low-boiling and nucleophilic solvents, such as water, to high-boiling and largely nonpolar solvents is suspected to affect the chemistry of the liquefaction process [57]. Low-molecular-weight cosolvents such as phenol, propanol, ethanol, methanol, and glycerol are applied and preferable in order to obtain low-molecular-weight product compounds. Polar protic solvents gave higher conversion rates and bio-oil/gas yields [58].

Methanol is an excellent solvent for cellulose liquefaction due to its high polarity, which allows penetration even into crystalline cellulose regions, promoting the solvolysis of polymer chains [31,59] with a maximum bio-oil yield varying from 32.21% [31] up to 100% [60], but the reaction temperature and pressure must be quite high due to its physical properties [31].

Glycerol was found to be a good solvent for cellulose liquefaction because its polar molecules can easily penetrate into the cellulose structure. Furthermore, glycerol liquefaction can be performed at high temperatures at atmospheric pressure [61].

Some solvents such as formic acid, light alcohols such as 2-propanol, and hydrocarbons such as tetralin are hydrogen donor solvents. These solvents effectively stabilize liquefaction products which helps to reduce product repolymerization [56]. Compared to non-hydrogen donor solvents, hydrogen donor solvents show significant improvement in cellulose conversion and in the bio-oil quality [62].

Ionic liquids are also efficient for cellulose liquefaction [63] due to their high thermal and chemical stability, high polarity, low melting points, and good cellulose solvating properties [64]. Cellulose liquefaction in ionic liquids is performed at 90–130 °C and ambient pressure, followed by addition of water and washing of the precipitate [65].

**Table 1:** Solvent used for biomass/cellulose HTL.

Solvent	Conversion (%)	Temperature (°C)	Ref.
NaOH/H_2_O	28–35	350	[66]
NaOH/H_2_O	77	120–250	[67]
Urea/H_2_O		100	[68]
H_2_SO_4_/H_2_O	85	150	[42]
H_3_PO_4_/H_2_O	85	150	[69]
Citric acid/H_2_O	65	100	[68]
Oxalic acid/H_2_O	65	100	[68]
Glycerol	60	100	[68]
Iso-propanol		380	[70]
2-propanol	32–49	240–320	[71]
2-butanol	27–53	240–320	[71]
Ethanol	100	100–250	[60]
Methanol	100	100–250	[60]
1-octanol		270	[72]
Acetone	60.5	299	[73]
Polyethylene glycol		150	[74]
Phenol		130–150	[74]
Dimethylsulfoxide	100		[75]
Ethylacetate	100		[76]
[Alkylmethylimidazolium]Cl	75–90	120	[60]
[Bmim]Cl	20	100	[77]
[Bmim]Ac	11.5	50	[77]
[Amin]Cl	3.5	100	[78]
[Emim]Cl	10	100	[77]

### 2.5. Other Decomposition Processes

Cellulose exposed to microwave irradiation (frequencies 0.3 to 300 GHz and wavelength from 1 mm to 1 m) begins to decompose due to local heating [79]. Microwave heating in cellulose liquefaction leads to a reduction in time and energy consumption [80] and allows effective dissolution and depolymerization under mild reaction conditions [61,81,82].

Microwave liquefaction of microcrystalline cellulose in water under acidic conditions produces mainly 5-hydroxymethylfurfural and levulinic acid. At 120 °C and 150 °C, almost 100% selectivity was observed. At 180 °C, less selectivity was observed and glucose appeared in the product mixture [45].

The sedimentation of particles in the liquid medium, which occurs even at normal earth gravity (1 G), can be accelerated by an ultracentrifuge machine. Strong acceleration of the gravitation field (more than 10^6^ G) can induce a decomposition reaction that, in combination with hydrothermal decomposition, results in cellulose decomposition. Under a strong gravitation field, glucose and its decomposition products exist in the acidic media around the capsule [83].

Crystalline cellulose contains an ordered hydrogen bonded network within which a proton transport network is possible in the presence of an electromagnetic field [84]. Ultrasonic waves are found to intensify physical and chemical processes, such as extraction and hydrolysis, in treated materials, such as cellulose, resulting from the cavitation, mechanical, and thermal effects of the ultrasound [85].

## 3. Cellulose Dissolution

Cellulose dissolution is a process in which solvent molecules penetrate the cellulose structure and cause polymer swelling to certain extent. The physical properties and volume of the cellulose are significantly altered, while the chemical structure of the polymer remains practically unchanged [86]. Since cellulose is a polar molecule with a high degree of crystallinity [87], it has good hydrogen bonding ability [88].

**Figure 4:**
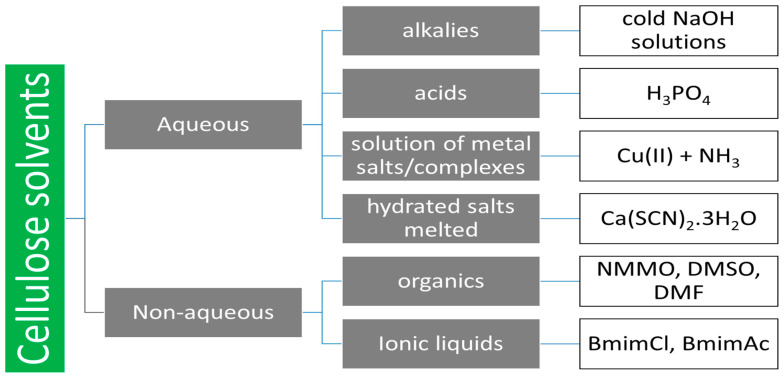
Cellulose solvents.

The dissolution of cellulose without chemical modification is difficult due to the long rigid cellulose chains and strong intermolecular and intramolecular hydrogen bonds [89] and hydrophobic interactions [90]. The common solvents applicable for the dissolution of cellulose are summarized in Figure 4.

It is not possible to define an ideal cellulose solvent because it is highly dependent on the purpose for which the cellulose is dissolved. Even if we focus only on the preparation of porous carbons, the selection of suitable solvent is obviously affected by the expected further processing of the solution, the possible use of crosslinkers, templates, or other additives, required physical form of regenerated cellulose (powder, fiber, monolith) and its behavior during subsequent carbonization.

Traditional processes of cellulose dissolution are based on the conversion of cellulose to water-soluble cellulose derivatives. The classic cellulose dissolution is a viscose process (using carbon disulfide) that is capable of preparing 8–12 wt% cellulose xanthogenate [14]. Recently, a new Lyocell process [91], using N-methylmorpholine-N-oxide (NMMO), was implemented [92,93]. The dissolution of cellulose in NMMO can be improved by microwave heating. Cellulose solutions in NMMO can be used for the preparation of cellulose membranes with a high degree of crystallinity [94]. However, new processes that use nontoxic chemicals and less energy than conventional technologies and produce a solution of cellulose (and not the cellulose derivatives) have to be developed.

Non-derivatizing cellulose solvents are copper hydroxide in ammonia or ethylenediamine solution and its alternatives, cadmium hydroxide or nickel oxide in the same aqueous ethylenediamine [95,96]. Although the industrial application of these systems is limited because of the heavy metals and their general environmental and health risks, conversion of cellulose dissolved together with metal ions to porous carbon containing metal or metal oxide (nano)particles can be interesting for electrochemical or catalytic applications. A similar system for the dissolution of cellulose, ferric chloride, tartaric acid dihydrate, and NaOH in cold water has been developed [97].

The dissolution of cellulose in aqueous media can be understood as an acid–base process where cellulose can act as an acid or as a base [95].

In addition to cellulose liquefaction in a hot acid solution under high pressure, concentrated phosphoric acid (85 wt%) can be used for the dissolution of cellulose [98]; however, cellulose is derivatized to cellulose phosphonate. Low temperature (100–200 °C) hydrolysis in concentrated acid leads to a glucose solution with nearly 100% yield [31].

On the other hand, cellulose dissolves in a cold (below 268 K) 8–10 wt% aqueous NaOH solution [99]. Additives such as polyethylene glycol [100], urea [6,101], thiourea [102], or a combination of urea and thiourea are frequently used [103]. All these substances are inexpensive and practically non-toxic. The solubility of cellulose in aqueous NaOH/urea solution depends on temperature, molecular weight of cellulose, and crystallinity of cellulose [104]. For its dissolution, a pre-cooled solution (−12.6 °C) of 7 wt% NaOH and 12 wt% urea was used. As a result of a fast self-assembly process between small molecules (water, urea, OH^−^) and cellulose macromolecules [105], cellulose dissolution occurs quickly (within a few minutes) at low temperatures (around or below 0 °C) [99]. The solubility increases with decreasing polymerization, but no effect of crystallinity was observed. Urea prevents the reaggregation of cellulose molecules by accumulation near hydrophobic regions [106]. The cellulose solution in NaOH/urea can easily form a gel [107]. An 8 wt% solution of cellulose in aqueous NaOH can be converted to porous cellulose material by physical gelation, chemical crosslinking with epichlorohydrin, coagulation in water, and lyophilisation [108]. At low temperature, celluloses I, III, and IV are converted to cellulose II [6]. Cellulose I and cellulose II are the most common polymorphs in regenerated cellulose. The hydrogen bonding in cellulose II is more complex than in cellulose I, leading to different mechanical properties and hydrophilicity [109]. Cellulose II can be prepared by alkaline treatment and regeneration. Cellulose III can be formed from cellulose I and II by treatment with ammonia. Cellulose IV is obtained by heating cellulose III [110].

The solubility of cellulose in water under mild conditions is promoted by the addition of certain inorganic salt hydrates with a water to salt molar ratio equal or less than the coordination number of the cation [111]: a mixture of NaSCN and KSCN with Ca(SCN)_2_·3 H_2_O or DMSO is a good cellulose solvent [112]. Cellulose dissolution was also observed in concentrated (63% *w*/*w*) aqueous solution of zinc chloride [95].

Various non-aqueous systems have been tested for cellulose dissolution, such as the combination of SO_2_ and NH_3_ with suitable ammonium salt [113]; mixtures of polar organic liquid such as formamide, *N*,*N*-dimethylformamide, DMSO; *N*,*N*-dimethylacetamide, SO_2_ or SOCl_2_ and aliphatic or alicyclic amines [114]; mixtures of amine-containing component; polar organic liquid and inorganic salt such as NH_3_/NaCl/DMSO or ethylenediamine/NaI/DMF [115]; two-component mixtures such as NH_3_/NH_4_SCN [95]; thiocyanates with hydrazine, hydrazine hydrate or ethylenediamine [116]; DMSO with methylamine, KSCN, CaCl_2_, formaldehyde or substituted ammonium fluorides [115]. The last-mentioned type of mixture is capable of dissolving cellulose with a high degree of polymerization at room temperature without any pretreatment and in a couple of minutes [117]. Another relevant two-component cellulose solvent is the ethylenediamine/KSCN mixture [118]. For faster dissolution of cellulose under mild conditions, ionic liquids such as 1-butyl-3-methylimidazolium acetate are used in dimethylsulfoxide, in *N*,*N*-dimethylformamide or in *N*,*N*-dimethylacetamide [119,120,121]. No additional electrolyte is required [122]. Up to 10% by weight of cellulose can be dissolved in 1-butyl-3-methylimidazoilium chloride at 100 °C; solubility can be increased to 25 wt% by microwave heating.

When the mixture of *N*,*N*-dimethylacetamide with LiCl is used as a solvent, the cellulose solubility can be improved if the cellulose suspension in water is freeze-dried. This treatment has better results than hot drying [123]. This effect is associated with an increase in cellulose porosity after freeze-drying.

Cellulose can be regenerated from the solution in ionic liquid by adding an anti-solvent such as water, alcohol, or acetone [124], however, at lower concentrations (less than 15% by weight), water acts as a viscosity-reducing agent in the solution of cellulose in ionic liquid [125]. Miscibility of ionic liquid with water or organic solvents can be tuned by length of the cation side chain and choice of anion [126].

## 4. Porous Carbons Based on Liquefied/Dissolved Cellulose

The traditional way of production of porous carbons is direct carbonization of biosources with subsequent activation (activated carbons). Direct carbonization of biomass is still a very active topic intensively reported in the literature (e.g., [127]) because it represents a cheap and effective method of highly porous carbon production.

After the development of hydrothermal treatment of biomass for the production of biofuels, the same technique attracts attention as a tool for the porous carbons production of either biomass [128] or cellulose (e.g., [129]).

The above-mentioned approaches are based on solid–solid conversion, which is in principle problematic for controlling the porosity of produced carbons and does not enable involvement of recent ways of porosity formation and control like templating.

To overcome this disadvantage, phenol-based wood liquefaction under acidic catalysis was developed [130,131], leading to a phenolic solution suitable for formaldehyde condensation with formaldehyde (in the presence of a suitable template) to gel-like solids carbonizable to porous carbons. Because of the large excess of phenol used in this approach, produced carbons are mostly based on phenol-formaldehyde resin, and biomass-based phenolic compounds are only partly involved.

Surprisingly, there is a lack of information on the direct use of bio-oil for production of carbons with controlled porosity. Zhu et al. [132] simply carbonized heavy bio-oil and after activation with NaOH they received highly porous carbon with surface area (derived from BET) greater than 2000 m^2^/g and total pore volume 1.7 cm^3^/g. Similar materials were obtained with the use of crayfish shell as a biological template during carbonization of heavy bio-oil followed by washing with acid and activation [133]. Carbon materials with developed micro and mesoporosity were also prepared by carbonization of bio-oil in the presence of MgO [134] or CaO (introduced as acetate) [135] as hard templates washable with acidic media. Another approach was described by Wang et al. [136] which used bio-oil hydrothermal treatment in steel autoclave (170 °C, 10 h) in the presence of cetyltrimethylamoniumbromide and nickel nitrate, followed by carbonization and activation. They reported a positive effect of increasing the amount of CTAB on surface characteristics improvement (surface area up to 1300 m^2^/g, pore volume 0.84 cm^3^/g, mesopores with diameter up to 30 nm).

Cellulose is one of the most studied bioprecursors for production of porous carbons. In addition to cellulose itself, its derivatives such as acetyl- [137], carboxymethyl- [138,139], ethyl- [140], hydroxyethyl- [141], and hydroxypropylcellulose [142] were used for carbon preparation.

The basic approach to conversion of cellulose into carbons with controllable porosity is usually of cellulose (or its derivative) followed with solidification (gelation, regeneration, freezing) in the presence of an appropriate template (not always) and final carbonization (and possible activation). Micropores in the porous carbons of cellulose are generated during the carbonization of the gels, and are increased by activation as with NaOH or KOH [143]. Mesopores usually originate from hard or soft templates, but they can also be affected by activation step [144].

The porous carbons of carboxymethylcellulose were prepared by ice-templating and carbonization. The carboxymethylcellulose aqueous solution was simply frozen in a liquid nitrogen bath, dried in a freeze-dryer for some days, and carbonized [145]. With the dissolution of cellulose in the calcium thiocyanate/water/ethanol system and its carbonization, porous carbons were obtained [146]. Highly porous materials were also obtained by dissolution of cellulose in ionic liquid or in lithium chloride/*N*,*N*-dimethylacetamide and its precipitation in a coagulation bath of water [147].

Another approach of cellulose gel preparation is the freeze–thaw process. In general, cellulose monoliths can be prepared by the freeze–thaw process of alkaline cellulose solution with NaOH, KOH, or in mixture with urea/thiourea [148]. The freezing and heating of dissolved cellulose solution at room temperature leads to the formation of cellulosic gels [149]. Cellulose gels have also been prepared by freezing solutions of cellulose Ca(SCN)_2_/water solvent [150].

Cellulose porous monoliths have also been prepared by controlled freezing of aqueous cellulose solution in a liquid nitrogen bath. Monolithic carbon was formed after carbonization [151]. Pluronic F-127 has been used as a porogen agent during the hydrothermal treatment of cellulose suspension in water and the resulting char was consequently carbonized to porous monolithic carbon [152].

## 5. Conclusions

Cellulose represents a valuable source of carbon for the production of porous carbonaceous materials [153]. Obviously, the simplest way to convert cellulose to carbon is direct carbonization, but if we want to control the properties of the products (especially porosity), more advanced approaches to carbon synthesis are required, in general based on liquid carbon precursors [154].

The conversion of cellulose into the liquid state can be achieved in two ways: decomposition to liquid mixture of low-molecular-weight organics (hydrothermal liquefaction or other decomposition techniques) or dissolution in suitable solvent (cellulose or its derivatives).

In the first case, the obtained mixtures contain a large number of chemical individuals with different properties and have a variable composition that is significantly dependent on the liquefaction condition. To find a common method of controlled conversion of such a complicated system into carbon with specific porosity would be very difficult.

However, if dissolution of cellulose (or its derivative) is successful, the obtained solution is homogeneous with more or less constant properties of dissolved cellulose given by the used solvent. Such a system is much easier to a transform to defined macrostructure (e.g., by cross-linking, gelation, and templating), consequently leading to carbons with defined porous structure.

Although many pathways of cellulose liquefaction/dissolution have been developed, this area still presents a field for intensive research, especially in connection with increasing interest in carbon-based porous materials for various advanced applications.

The challenging research topics in the near future could be focused on (among other areas):

- Transformation of industrially available bio-oils towards their chemical stability and uniformity, enabling their conversion to nanostructured organic and consequently carbon matter.
- Avoiding the loss of carbon, increasing the carbon yield during biomass treatment, e.g., by suitable cellulose derivatization, more extensive involvement of lignin constituent of biomass, etc.
- Production of biomass- or cellulose-based carbon monoliths with hierarchical porosity is still a challenge even for carbons produced from petroleum-based chemicals.

## Data Availability

The data presented in this study are available on request from the corresponding author.
